# SGLT-2 inhibitors improve cardiovascular and renal outcomes in patients with CKD: a systematic review and meta-analysis

**DOI:** 10.1038/s41598-023-42989-z

**Published:** 2023-09-23

**Authors:** Thomas A. Mavrakanas, Michael A. Tsoukas, James M. Brophy, Abhinav Sharma, Karim Gariani

**Affiliations:** 1 Division of Nephrology, Department of Medicine, McGill University Health Center Research Institute|CORE|Office 2B.44, 5252 de Maisonneuve West, Montreal, QC H4A 3S9 Canada; 2 Division of Endocrinology, McGill University Health Centre, Montreal, QC Canada; 3 Division of Cardiology, McGill University Health Centre, Montreal, QC Canada; 4 Division of Endocrinology, Diabetes, Nutrition and Patient Therapeutic Education, Geneva University Hospitals, Geneva, GE Switzerland

**Keywords:** Cardiology, Endocrinology, Nephrology

## Abstract

The effect of sodium-glucose co-transporter-2 (SGLT-2) inhibitors on cardiovascular and renal outcomes has not been systematically reviewed across baseline kidney function groups. We conducted a systematic review and meta-analysis of randomized control trials (RCTs) with SGLT-2 inhibitors in patients with and without CKD. We performed a PubMed/Medline search of randomized, placebo-controlled, event-driven outcome trials of SGLT-2 inhibitors versus active or placebo control in patients with and without diabetes from inception to November 2022. CKD was defined as an estimated glomerular filtration rate (eGFR) < 60 ml/min/1.73m^2^ (PROSPERO registration CRD4202016054). The primary outcome was cardiovascular death. Secondary outcomes included hospitalization for heart failure, major adverse cardiovascular events, CKD progression, all-cause mortality, treatment discontinuation, and acute kidney injury (AKI). The relative risk (RR) was estimated using a random-effects model. Twelve RCTs were included in this meta-analysis (89,191 patients, including 38,949 with eGFR < 60 ml/min/1.73m^2^). Use of an SGLT-2 inhibitor in patients with CKD was associated with a lower incidence of cardiovascular death (RR 0.87; 95% CI 0.79–0.95) and of heart failure (RR 0.67; 95% CI 0.61–0.75), compared with placebo. Heart failure risk reduction with SGLT-2 inhibitors was larger among patients with CKD compared with patients without CKD (RR for the interaction 0.87, 95% CI 0.75–1.02, and p-value for interaction 0.08). SGLT-2 inhibitors were associated with a lower incidence of CKD progression among patients with pre-existing CKD: RR 0.77 (95% CI 0.68–0.88), compared with placebo. Among patients with CKD, a lower risk of AKI (RR 0.82; 95% CI 0.72–0.93) and treatment discontinuation was seen with SGLT-2 inhibitors compared with placebo. SGLT-2 inhibitors offer substantial protection against cardiovascular and renal outcomes in patients with CKD. These results strongly advocate in favor of using them in patients with CKD and keeping them as kidney function declines.

## Introduction

Sodium-glucose co-transporter-2 (SGLT-2) inhibitors are glucose-lowering drugs that act by reducing renal reabsorption of glucose at the S1 segment of the proximal tubule in the kidney. They induce glycosuria and natriuresis and are associated with reduction in glycated hemoglobin (Hb1Ac), blood pressure, albuminuria, and body weight^1,2^. The cardiovascular and renal benefits of SGLT-2 inhibitors have been demonstrated in several large randomized clinical trials (RCT) in patients with type 2 diabetes and more recently in patients without type 2 diabetes^3^. Assessing the magnitude of the protective effect of SGLT-2 inhibitors on these outcomes based on the presence or absence of chronic kidney disease (CKD) at baseline remains an important question in order to confirm whether their therapeutic effect is independent of kidney function^4^.

The aim of this study is to perform a systematic review and meta-analysis of RCTs assessing the effect of SGLT-2 inhibitors on cardiovascular and renal outcomes according to baseline CKD status in individuals with or without type 2 diabetes. This is the most recent meta-analysis on this important question, including the most recent clinical trials with SGLT-2 inhibitors.

## Methods

This systematic review and meta-analysis was conducted and reported using the Preferred Reporting Items for Systematic Reviews and Meta-Analysis (PRISMA) statement^5^. The protocol for this review was prospectively registered in the International Prospective Register of Systematic Reviews (PROSPERO registration number CRD42019131774) and can be accessed at: http://www.crd.york.ac.uk/PROSPERO/display_record.php?ID=CRD42019131774.

### Search strategy and selection criteria

We searched MEDLINE from inception to November 2022 to identify potentially eligible studies. The following search terms were used: ((((((myocardial infarction) OR (stroke or cerebrovascular accident)) OR (heart failure or cardiac failure)) OR (death OR mortality)) OR (“Cardiovascular Diseases”Mesh])) OR (kidney failure)) AND ((empagliflozin or canagliflozin or dapagliflozin or sotagliflozin or ertugliflozin or ipragliflozin or tofogliflozin or sergliflozin or remogliflozin or luseogliflozin) OR (“Sodium-Glucose Transporter 2 Inhibitors”[Mesh])). Search was limited to clinical trials or RCTs.

We included all randomized, placebo-controlled, event-driven outcome trials of SGLT-2 inhibitors versus active or placebo control. Trials including participants with type 1 diabetes or individuals < 18 years of age were excluded. Inclusion of patients with CKD was required. CKD was defined as an eGFR < 60 ml/min/1.73m^2^. RCTs had to be peer reviewed manuscripts with a minimum follow-up of 6 months. At least one of the following cardiovascular or renal outcomes had to be reported: cardiovascular death, hospitalization for heart failure, major adverse cardiovascular events, renal death, or CKD progression.

Two authors (K.G. and M.T.) independently screened the titles and abstracts of all identified articles and, when required, reviewed full-text manuscripts to identify potentially relevant studies. The reference lists of all selected studies and available meta-analyses were also reviewed to search for any additional qualifying studies. Any disagreement related to the identification or eligibility of studies was resolved through discussion with a third author (T.M.).

### Data synthesis and analysis

Two authors (T.M. and K.G.) independently extracted all relevant baseline characteristics and study outcomes using a standardized digital extraction form, including treatment effects in patient subgroups defined by the presence or absence CKD. Any discrepancies in data extraction or risk-of-bias assessment were resolved by consensus.

Efficacy outcomes of interest included: major adverse cardiovascular events (MACE), including cardiovascular death, nonfatal myocardial infarction, or nonfatal stroke; the composite of cardiovascular death or hospitalization for heart failure or their individual components, CKD progression (a composite outcome of persistent eGFR decline of at least 40% or renal replacement therapy initiation), and all-cause mortality. The primary outcome was cardiovascular mortality. We also extracted information on treatment discontinuation and acute kidney injury. A detailed definition of clinical outcomes in each trial included in this meta-analysis is depicted in Table 1.

**Table 1:** Outcome definitions.

Study	Definition of MACE	Definition of composite CV outcome	Definition of renal outcome	All-cause mortality	CV death	Heart failure
EMPA-REG outcome	Death from CV causes, nonfatal myocardial infarction or nonfatal stroke		Macroalbuminuria, doubling creatinine, ESKD, renal death	X	X	X
CANVAS	Death from CV causes, nonfatal myocardial infarction or nonfatal stroke	CV death or hospitalization for heart failure	≥ 40% GFR decline, ESKD, renal death		X	X
DECLARE-TIMI 58	CV death, myocardial infarction, or ischemic stroke	CV death or hospitalization for heart failure	≥ 40% GFR decline, ESKD, renal death	X	X	X
CREDENCE	Death from CV causes, nonfatal myocardial infarction or nonfatal stroke	CV death or hospitalization for heart failure	Doubling creatinine, ESKD, renal or CV death		X	X
DAPA-CKD		CV death or hospitalization for heart failure	≥ 50% GFR decline, ESKD, renal or CV death	X	X	
DAPA-HF		CV death or HF hospitalization/urgent HF visit	≥ 50% GFR decline, ESKD, renal or CV death	X	X	X
VERTIS-CV	Death from CV causes, nonfatal myocardial infarction, or nonfatal stroke	CV death or hospitalization for heart failure	Doubling creatinine, ESKD, renal death		X	X
SCORED	Death from CV causes, nonfatal myocardial infarction, or nonfatal stroke	CV death or HF hospitalization/urgent HF visit	≥ 50% GFR decline, ESKD, renal death	X	X	X
EMPEROR-REDUCED		CV death or hospitalization for heart failure	≥ 40% GFR decline, ESKD	X	X	
EMPEROR-PRESERVED		CV death or hospitalization for heart failure				
DELIVER		CV death or HF hospitalization/urgent HF visit	≥ 50% GFR decline, ESKD, renal death			
EMPA-KIDNEY		CV death or hospitalization for heart failure	≥ 40% GFR decline, ESKD, renal death	X	X	

MACE, major adverse cardiovascular event; CV, cardiovascular; ESKD, end-stage kidney disease; GFR, glomerular filtration rate.

CKD was defined as an eGFR < 60 ml/min/1.73m^2^ in most studies. In the DAPA-CKD trial, patients with a urine albumin to creatinine ratio (UACR) of 200–5000 mg/g and an eGFR as high as 75 ml/min/1.73m^2^ were included^3^. Similarly, in the EMPEROR-Reduced and EMPEROR-Preserved studies, CKD was defined as eGFR < 60 ml/min/1.73m^2^ or a UACR > 300 mg/g for the outcomes of cardiovascular death, all-cause mortality, or for the composite renal outcome^6,7^. In EMPA-KIDNEY, CKD was defined as eGFR < 45 ml/min/1.73m^2^ regardless of the level of albuminuria or a UACR > 200 mg/g and an eGFR of 45–89 ml/min/1.73m^2^^8^.

### Statistical analysis

The relative risk (RR) with associated 95% confidence intervals was the principal summary measure. When the number of events per group was not reported, RR were calculated from the incidence rates, the hazard ratio, and the total number of events and participants^9^.

The pooled RR for each outcome was estimated using a random-effects model with the standard DerSimonian & Laird approach^10^. Results were presented in a Forest plot. Prediction intervals were also reported. Prediction intervals reflect the effect to be expected in future patients and their use in meta-analyses has been strongly advocated by prominent scholars in the field^11^.

Interaction effects were estimated to test for treatment effect modification by CKD status. Only studies reporting outcomes in patients with and without CKD were included for interaction terms calculation. The natural logarithm of the relative risk ratio and the standard error of the natural logarithm of the relative risk were pooled using the inverse variance method and a random effects model.

For study quality assessment (performed by K.G. and M.T.), the second version of the Cochrane Risk-of-bias tool for RCTs (RoB2) was used^12^.

The I^2^ index was used to quantify heterogeneity and assess inconsistency. A funnel plot was drawn to assess publication bias. Heterogeneity was considered to be low, moderate, or high if *I*^*2*^ was less than 25%, 26% to 75%, or greater than 75%, respectively^13^.

The GRADE approach was used to rate confidence in effect estimates^14^. The initial rating for RCTs was high and decreased in presence of serious inconsistency, indirectness, imprecision, risk of bias, or when publication bias was likely.

We used the risk in the placebo group, along with the pooled relative risk for overall patients at long-term follow-up from the systematic review, to calculate the absolute effect estimates in our evidence summaries.

Statistical analyses were performed in Stata (version 17 SE; College Station, TX). Risk of bias plots were created using the *robvis* tool^15^.

## Results

### Study characteristics

A total of 405 articles were identified. After title and abstract screening, 41 articles were selected for full-text review. Twelve RCTs (19 publications) were identified and included in this meta-analysis (Supplemental Fig. 1)^3,8,16–32^. These studies enrolled a total of 89,191 patients, including 38,949 with an eGFR < 60 ml/min/1.73m^2^. Study characteristics for the included trials are shown in Table 2. For this Table, information from the main publication for each trial was used. For some of the outcomes mentioned below, data from subsequent publications was used and this explains minor differences in the denominator for these outcomes.

**Table 2:** Study characteristics.

Study	Type of SGLT-2 (dose, mg)	Number of participants	Median follow-up, years	Age, years	Women (%)	ACEI- ARB at baseline (%)	uACR > 30 mg/g	Diabetes (%)	Baseline eGFR < 60*	Established CV disease	History of heart failure
EMPA-REG outcome	Empagliflozin (10 and 25)	7020	3.1	63 (8.7)	28.5	5666 (80.7%)	2782 (40.0)	7020 (100)	1819 (25.9)	7020 (100)	706 (10.1)
CANVAS	Canagliflozin (100 and 300)	10,142	2.4	63.3 (8.3)	35.8	8116 (80%)	3026 (30.0)	10,142 (100)	2039 (20.1)	6656 (65.6)	1461 (14.4)
DECLARE-TIMI 58	Dapagliflozin (10)	17,160	4.2	63.9 (6.8)	37.4	13,950 (81.3%)	5199 (30.3)	17,160 (100)	1265 (7.4)	6974 (40.6)	1724 (10.0)
CREDENCE	Canagliflozin (100)	4401	2.6	63.0 (9.2)	33.9	4395 (99.9%)	4401 (100)	4401 (100)	2592 (58.9)	2223 (50.5)	652 (14.8)
DAPA-CKD	Dapagliflozin (10)	4304	2.4	61.85 (12.1)	33.1	4224 (98.1%)	4304 (100)	2906 (67.5)	3850 (89.5)	1610 (37.4)	468 (10.9)
DAPA-HF	Dapagliflozin (10)	4744	1.5	66.3 (10.9)	23.4	4460 (94%)	NA	1983 (41.8)	1926 (40.6)	4744 (100)	4744 (100)
VERTIS-CV	Ertugliflozin (5 and 15)	8246	3.5	64.4 (8.05)	29.9	6686 (81.1%)	3247 (39.4)	8246 (100)	1807 (21.9)	8238 (99.9)	1958 (23.7)
SCORED	Sotagliflozin (200 to 400)	10,584	1.33	69 (NA)	44.9	9229 (87.2%)	6875 (65.0)	10,584 (100)	10,584 (100)	NA	3283 (31.0)
EMPEROR-REDUCED	Empagliflozin (10)	3730	1.33	66.85 (11)	23.9	3327 (89.2%)	1632 (43.8)	1856 (49.8)	1799 (48.2)	3730 (100)	3730 (100)
EMPEROR-PRESERVED	Empagliflozin (10)	5988	2.2	71.9 (9.6)	44.7	4832 (80.7%)	NA	2938 (49.1)	2988 (49.9)	5988 (100)	5988 (100)
DELIVER	Dapagliflozin (10)	6263	2.3	71.7 (9.6)	43.9	2272 (36.3%)	NA	2806 (44.8%)	3070 (49.0)	6263 (100)	6263 (100)
EMPA-KIDNEY	Empagliflozin (10)	6609	2.0	63.9 (13.9)	33.2	5628 (85.2%)	5281 (79.9%)	3040 (46.0%)	5210 (78.8)*	1765 (26.7)	NA

Results are presented as mean (standard deviation) or number (percentage). ACEI, angiotensin-converting enzyme inhibitor; ARB, angiotensin receptor blocker; NA, not available; uACR, urine albumin-creatinine ratio; eGFR, estimated glomerular filtration rate (in ml/min/1.73m^2^); CV, cardiovascular.

*eGFR < 45 ml/min/1.73m2 for the EMPA-KIDNEY trial.

Five SGLT-2 inhibitors, canagliflozin, dapagliflozin, empagliflozin, ertugliflozin, and sotagliflozin were used. Most trials enrolled patients with a minimum eGFR of 30 ml/min/1.73m^2^ at baseline. The DAPA-CKD, SCORED, and DELIVER studies allowed for participant enrolment with an eGFR ≥ 25 ml/min/1.73m^2^^3,27,32^, while the EMPEROR-Preserved, Reduced, and EMPA-KIDNEY trials with an eGFR ≥ 20 ml/min/1.73m^2^^8,25,26^.

Five studies had formal discontinuation criteria based on kidney function. The cutoff of 15 and 30 ml/min/1.73m^2^ was used in the CANVAS and DECLARE TIMI-58 trial, respectively^17,22^. In CREDENCE, participants were allowed to stay on the SGLT-2 inhibitor until dialysis initiation^21^. In VERTIS-CV and SCORED, the SGLT-2 inhibitor was discontinued when eGFR dropped below 15 ml/min/1.73m^2^ or when renal replacement therapy was required^23,27^.

Overall risk of bias was considered to be low (“not serious”) for all RCTs included in this meta-analysis (Supplemental Fig. 2). There was no major publication bias for any of the outcomes identified at the inspection of the funnel plots (Supplemental Fig. 3).

### Cardiovascular outcomes

#### Cardiovascular death

**Figure 1:**
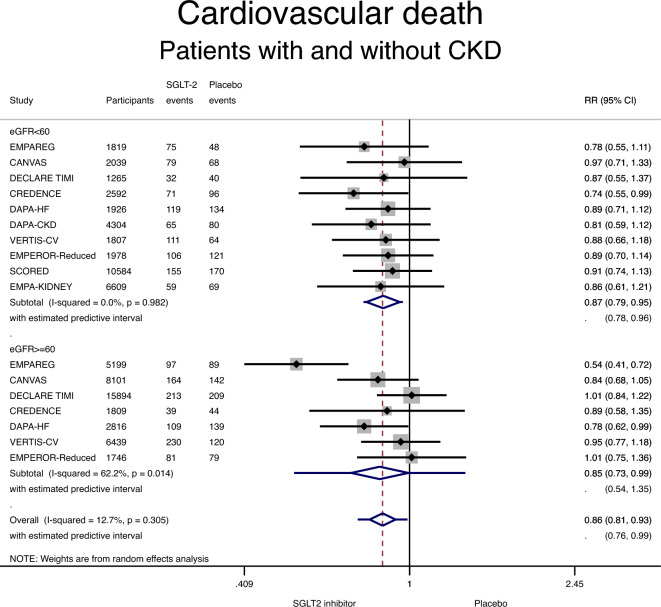
Forest plot showing the incidence of cardiovascular death with SGLT-2 inhibitors compared with placebo in patients with and without chronic kidney disease (CKD). Results are stratified by CKD status. Data are presented as risk ratios (RR) with 95% confidence intervals (95% CI). A lower incidence of cardiovascular death is identified with SGLT-2 inhibitors compared with placebo in patients with and without CKD (p for interaction 0.72). A random effects model is used.

Use of an SGLT-2 inhibitor was associated with a lower incidence of cardiovascular death in patients with CKD, compared with placebo: RR 0.87 (95% CI 0.79–0.95; P = 0.003) (Fig. 1 and Table 3). Similarly, use of an SGLT-2 inhibitor was associated with a lower incidence of cardiovascular death in patients without CKD: RR 0.85 (95% CI 0.73–0.99; P = 0.04) (Fig. 1 and Table 3). Significant heterogeneity was detected in the non-CKD group (I^2^ = 62%) but not in the CKD group (I^2^ = 0%). There was no interaction between CKD status and the effect of SGLT-2 inhibitors on cardiovascular death (RR 1.03, 95% CI 0.88–1.20; p for interaction 0.72).

**Table 3:** Clinical outcomes by treatment strategy and certainty of evidence.

Outcome	Study group	SGLT-2 inhibitor vs. placebo	Absolute effect estimate	Quality of evidence
CV death	CKD	RR 0.87 (95% CI 0.79–0.95)	7 fewer per 1000 (95% CI 3 fewer–11 fewer)	HIGH
	Non- CKD	RR 0.85 (95% CI 0.73–0.99)	7 fewer per 1000 (95% CI 0 fewer–12 fewer)	MODERATE^a^ Serious inconsistency
Heart Failure	CKD	RR 0.67 (95% CI 0.61–0.75)	29 fewer per 1000 (95% CI 22 fewer–34 fewer)	HIGH
	Non- CKD	RR 0.78 (95% CI 0.70–0.86)	9 fewer per 1000 (95% CI 6 fewer–12 fewer)	HIGH
CV death or heart failure	CKD	RR 0.79 (95% CI 0.74–0.84)	25 fewer per 1000 (95% CI 19 fewer–31 fewer)	HIGH
	Non- CKD	RR 0.84 (95% CI 0.77–0.91)	14 fewer per 1000 (95% CI 8 fewer–20 fewer)	HIGH
MACE	CKD	RR 0.84 (95% CI 0.75–0.94)	18 fewer per 1000 (95% CI 7 fewer–28 fewer)	HIGH
	Non- CKD	RR 0.93 (95% CI 0.87–0.99)	7 fewer per 1000 (95% CI 1 fewer–12 fewer)	HIGH
Renal outcome	CKD	RR 0.77 (95% CI 0.68–0.88)	18 fewer per 1000 (95% CI 10 fewer–26 fewer)	MODERATE^b^ Serious indirectness
	Non- CKD	RR 0.65 (95% CI 0.53–0.80)	8 fewer per 1000 (95% CI 5 fewer–11 fewer)	MODERATE^b^ Serious indirectness
All-cause mortality	CKD	RR 0.87 (95% CI 0.80–0.95)	10 fewer per 1000 (95% CI 4 fewer–15 fewer)	HIGH
	Non- CKD	RR 0.84 (95% CI 0.69–1.01)	12 fewer per 1000 (95% CI 22 fewer–1 more)	LOW^c^ Serious imprecision & inconsistency
Treatment discontinuation	CKD	RR 0.88 (95% CI 0.82–0.95)	23 fewer per 1000 (95% CI 10 fewer–35 fewer)	HIGH
	Non- CKD	RR 1.59 (95% CI 0.60–4.19)	54 more per 1000 (95% CI 37 fewer–294 more)	VERY LOW^d^ Very serious imprecision & inconsistency
Acute kidney injury	CKD	RR 0.82 (95% CI 0.72–0.93)	11 fewer per 1000 (95% CI 17 fewer–4 fewer)	HIGH
	Non- CKD	RR 1.19 (95% CI 0.71–2.01)	4 more per 1000 (95% CI 7 fewer–23 more)	VERY LOW^e^ Very serious imprecision & serious inconsistency

SGLT-2, sodium-glucose co-transporter-2; CKD, chronic kidney disease; RR, relative risk; CI, confidence interval; CV, cardiovascular; MACE, major adverse cardiovascular events.

^a^Serious inconsistency: I^2^ = 62%.

^b^Serious indirectness: variable definition of the composite renal outcome (some of the studies included cardiovascular death in this outcome).

^c^Serious imprecision: confidence interval includes no difference. Serious inconsistency: I^2^ = 71%.

^d^Very serious imprecision: wide confidence interval that includes no difference. Very serious inconsistency: I^2^ = 99%.

^e^Very serious imprecision: wide confidence interval includes no difference. Serious inconsistency: I^2^ = 76%.

#### Heart failure

**Figure 2:**
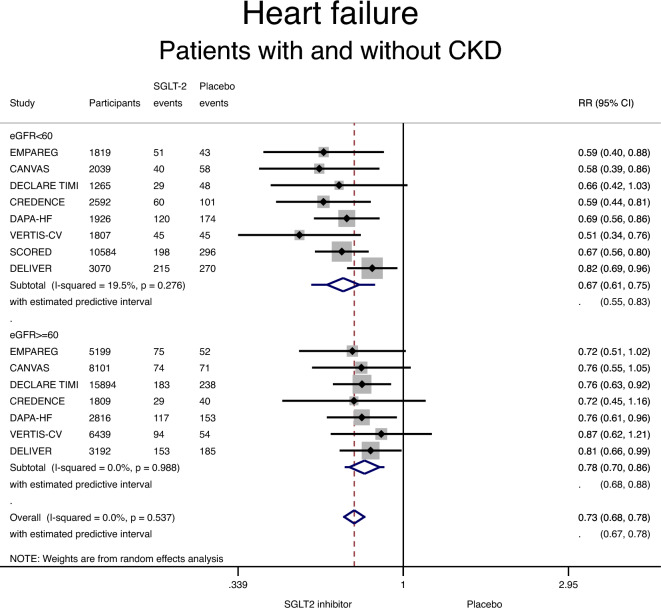
Forest plot showing the incidence of heart failure with SGLT-2 inhibitors compared with placebo in patients with and without chronic kidney disease (CKD). Results are stratified by CKD status. Data are presented as risk ratios (RR) with 95% confidence intervals (95% CI). A lower incidence of heart failure is identified with SGLT-2 inhibitors compared with placebo in patients with and without CKD (p for interaction 0.08 suggesting a stronger treatment effect with SGLT-2 inhibitors in the subgroup of patients with CKD). A random effects model is used.

Use of an SGLT-2 inhibitor was associated with a lower incidence of heart failure in patients with CKD, compared with placebo: RR 0.67 (95% CI 0.61–0.75; P < 0.001) (Fig. 2 and Table 3). Moreover, use of an SGLT-2 inhibitor was associated with a lower incidence of heart failure in patients without CKD: RR 0.78 (95% CI 0.70–0.86; P < 0.001) (Fig. 2 and Table 3). No significant heterogeneity was detected in the non-CKD or the CKD subgroup (I^2^ of 0% and 20%, respectively). A significant interaction was detected between CKD status and the effect of SGLT-2 inhibitors on heart failure (RR 0.87, 95% CI 0.75–1.02, p for interaction 0.08): risk reduction with SGLT-2 inhibitors was more important among patients with CKD (Table 3).

#### Cardiovascular death or heart failure

SGLT-2 inhibitors were associated with a lower incidence of cardiovascular death or hospitalization for heart failure among patients with CKD, compared with placebo: RR 0.79 (95% CI 0.74–0.84; P < 0.001) (Supplemental Fig. 4 and Table 3). Similarly, use of an SGLT-2 inhibitor was associated with a lower incidence of cardiovascular death or heart failure in patients without CKD: RR 0.84 (95% CI 0.77–0.91; P < 0.001) (Supplemental Fig. 4 and Table 3). Low or no heterogeneity was detected in the non-CKD and the CKD subgroup, respectively (I^2^ of 14% and 0%). There was no interaction between CKD status and the effect of SGLT-2 inhibitors on cardiovascular death or heart failure (RR 0.97, 95% CI 0.87–1.08, p for interaction 0.54).

#### MACE

**Figure 3:**
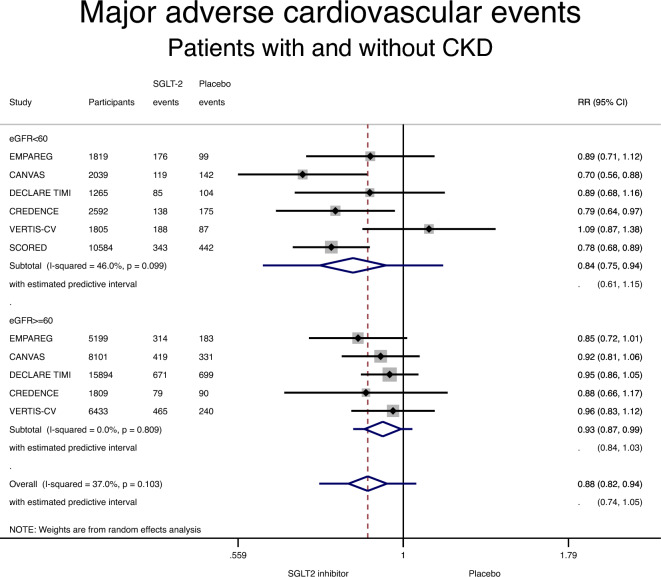
Forest plot showing the incidence of major adverse cardiovascular events (MACE) with SGLT-2 inhibitors compared with placebo in patients with and without chronic kidney disease (CKD). Results are stratified by CKD status. Data are presented as risk ratios (RR) with 95% confidence intervals (95% CI). A lower incidence of MACE is identified with SGLT-2 inhibitors compared with placebo in patients with and without CKD (p for interaction 0.44). A random effects model is used. Definition of MACE is detailed in Table 1.

We only included studies using the 3-point MACE definition (CV death, non-fatal myocardial infarction, or stroke). SGLT-2 inhibitors were associated with a lower incidence of MACE in patients with CKD, compared with placebo: RR 0.84 (95% CI 0.75–0.94; P = 0.003) (Fig. 3 and Table 3). Use of an SGLT-2 inhibitor was also associated with a lower incidence of MACE in patients without CKD: RR 0.93 (95% CI 0.87–0.99; P = 0.03) (Fig. 3 and Table 3). Moderate and no heterogeneity was detected in the CKD and the non-CKD group, respectively (I^2^ of 46% and 0%). There was no interaction between CKD status and the effect of SGLT-2 inhibitors on MACE (RR 0.95, 95% CI 0.82–1.09, p for interaction 0.44).

### Renal outcomes

**Figure 4:**
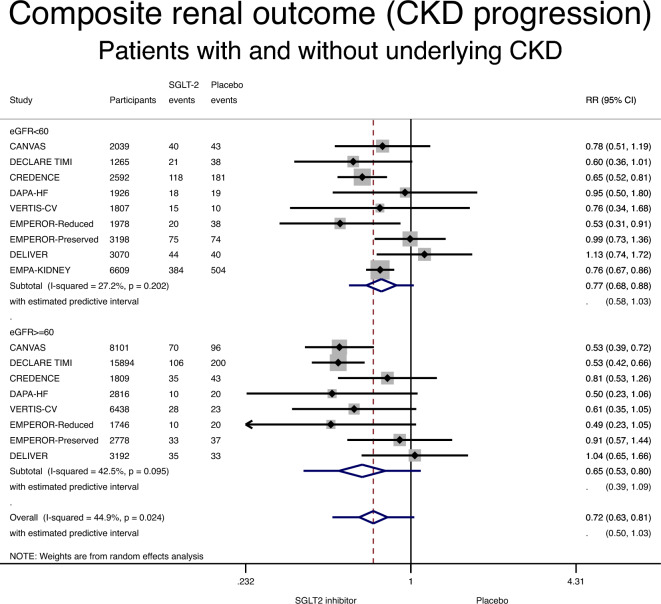
Forest plot showing the incidence of the composite renal outcome with SGLT-2 inhibitors compared with placebo in patients with and without chronic kidney disease (CKD). Results are stratified by CKD status. Data are presented as risk ratios (RR) with 95% confidence intervals (95% CI). A lower incidence of the composite renal outcome is identified with SGLT-2 inhibitors compared with placebo in patients with and without CKD (p for interaction 0.30). A random effects model is used. Definition of the composite renal outcome is detailed in Table 1.

Definition of the composite renal outcome was highly variable across different studies. In addition to variable percentages of eGFR decline and incidence of kidney failure, some of the trials grouped together cardiovascular death with death from renal cause and with CKD progression (Table 1). SGLT-2 inhibitors were associated with a lower incidence of the composite renal outcome in patients with CKD, compared with placebo: RR 0.77 (95% CI 0.68–0.88; P < 0.001) (Fig. 4 and Table 3). Similarly, use of an SGLT-2 inhibitor was associated with a lower incidence of the composite renal outcome in patients without CKD: RR 0.65 (95% CI 0.53–0.80; P < 0.001) (Fig. 4 and Table 3). Low and moderate heterogeneity were detected in the CKD and the non-CKD group (I^2^ of 27% and 43%), respectively. There was no interaction between CKD status and the effect of SGLT-2 inhibitors on the composite renal outcome (RR 1.13, 95% CI 0.90–1.41, p for interaction 0.30).

### All-cause mortality

SGLT-2 inhibitors were associated with a lower incidence of death from any cause among patients with CKD, compared with placebo: RR 0.87 (95% CI 0.80–0.95; P = 0.003) (Supplemental Fig. 5 and Table 3). However, the incidence of all-cause mortality was similar in the SGLT-2 inhibitor and placebo arms in patients without CKD: RR 0.84 (95% CI 0.69–1.01; P = 0.06) (Supplemental Fig. 5 and Table 3). Heterogeneity was low in the CKD group but high in the non-CKD group (I^2^ of 12% and 71%, respectively). There was no interaction between CKD status and the effect of SGLT-2 inhibitors on all-cause mortality (RR 1.02, 95% CI 0.87–1.20, p for interaction 0.79).

### Treatment discontinuation and acute kidney injury

Four studies examined treatment discontinuation by CKD status at baseline^8,16,20,25^. Among patients with CKD, treatment discontinuation was less commonly observed in the SGLT-2 inhibitor arm compared with the placebo arm: RR 0.88 (95% CI 0.82–0.95; P = 0.001) (Supplemental Fig. 6 and Table 3). In contrast, the incidence of treatment discontinuation was similar in both arms among patients without CKD: RR 1.59 (95% CI 0.60–4.19; P = 0.35). However, no interaction was identified between CKD status and the effect of SGLT-2 inhibitors on treatment discontinuation (RR 0.57, 95% CI 0.20–1.59, p for interaction 0.28). Heterogeneity was low in the CKD group but very high in the non-CKD group (I^2^ of 0% and 99%, respectively).

**Figure 5:**
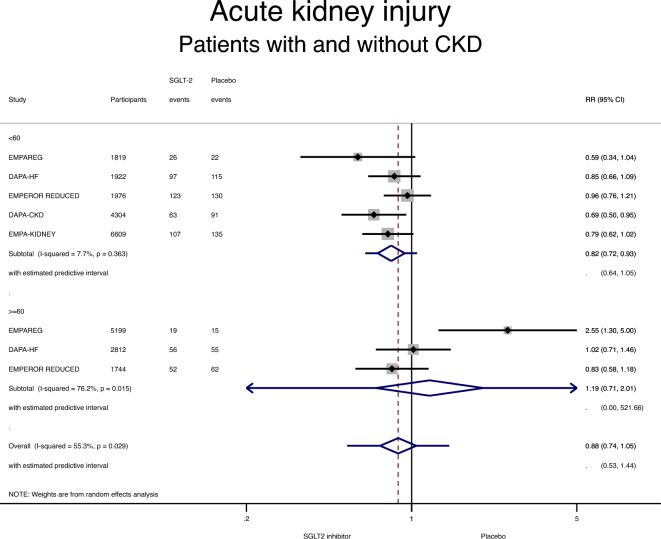
Forest plot showing the incidence of the acute kidney injury with SGLT-2 inhibitors compared with placebo in patients with and without chronic kidney disease (CKD). Results are stratified by CKD status. Data are presented as risk ratios (RR) with 95% confidence intervals (95% CI). A lower incidence of the acute kidney injury is identified with SGLT-2 inhibitors compared with placebo in patients with CKD. A random effects model is used.

Five studies examined acute kidney injury (AKI) by CKD status at baseline^3,8,16,20,25^. Among patients with CKD, AKI was less commonly observed in the SGLT-2 inhibitor arm compared with the placebo arm: RR 0.82 (95% CI 0.72–0.93; P = 0.003) (Fig. 5 and Table 3). In contrast, the incidence of AKI was similar in both arms among patients without CKD: RR 1.19 (95% CI 0.71–2.01; P = 0.51). However, no interaction was identified between CKD status and the effect of SGLT-2 inhibitors on AKI (RR 0.68, 95% CI 0.33–1.39, p for interaction 0.28). Heterogeneity was low in the CKD group but high in the non-CKD group (I^2^ of 8% and 76%, respectively).

Episodes of euglycemic ketoacidosis were rare and not reported by CKD status in the included trials.

## Discussion

This meta-analysis of RCTs examines cardiovascular and renal outcomes in patients with and without CKD and includes results from the most recently published clinical trials with SGLT-2 inhibitors in patients with and without diabetes. We show that the protective effect of SGLT-2 inhibitors is maintained as kidney function declines and that, due to the high baseline risk in patients with CKD, the absolute risk reduction with this treatment is greater in these patients. In addition, we show that the efficacy of these agents in reducing heart failure events is greater in patients with CKD, compared with patients with preserved renal function. These findings strongly support use of SGLT-2 inhibitors in patients with an eGFR < 60 ml/min/1.73m^2^.

When SGLT-2 inhibitors were introduced in clinical practice, they were not recommended in patients with CKD because of concerns of reduced efficacy in this setting. In this report, we present evidence that the cardiovascular and kidney benefits of these agents are maintained, if not reinforced, in patients with an eGFR at least as low as 30 ml/min/1.73m^2^. Could these agents be used in patients with lower eGFRs? Five of the trials included in this analysis had formal discontinuation criteria. Three of them used these agents in patients with an eGFR as low as 15 ml/min/1.73m^2^, while in one trial (CREDENCE) canagliflozin was continued until dialysis initiation^21^. Dapagliflozin was initiated with an eGFR as low as 25 ml/min/1.73m^2^ and empagliflozin with an eGFR of 20 ml/min/1.73m^2^^3,25,26^. We did not observe any safety signals and the benefit of SGLT-2i on CKD progression was maintained in these studies. In addition, rates of treatment discontinuation were lower in the SGLT-2 inhibitor arm compared with the placebo arm among patients with CKD. However, the exact reason for discontinuation was unfortunately not reported. Finally, dapagliflozin was found to be associated with lower incidence of abrupt renal function decline, defined as doubling of serum creatinine from most recent value, suggesting a protective effect against AKI^30^. Future studies should examine if these benefits are maintained when CKD progresses to kidney failure.

Heterogeneity was low for most outcomes, with the notable exception of cardiovascular death, death from any cause, or treatment discontinuation in the non-CKD subgroup. For these analyses, heterogeneity was driven by the point estimates from the EMPA-REG OUTCOME trial^15^. This study only enrolled patients with established cardiovascular disease that may not get the same benefit from SGLT-2 inhibitors as patients at risk for cardiovascular disease. In addition, this study was performed in an era when the hemodynamic and renal effects of SGLT-2 inhibitors were not widely known and this might have influenced discontinuation rates with this agent in patients without CKD who were thought to develop acute kidney injury when exposed to empagliflozin.

Based on these findings, we suggest using SGLT-2 inhibitors for cardiovascular and renal protection in patients with CKD and maintaining this treatment as kidney function declines until initiation of renal replacement therapy. Use in patients with severely reduced eGFR (stage V CKD) or on dialysis is not recommended at this time and requires further study. Careful monitoring of kidney function in patients experiencing acute events, such as infection or dehydration, is of utmost importance and use of a sick day rule is critical for patient education^33^. Furthermore, the effect of patient compliance and understanding of these rules on real-world clinical outcomes has not been adequately studied. However, reasonable concerns about rare side-effects should not preclude using these agents in patients with CKD. It is very important for the nephrology community to advocate for higher uptake of SGLT-2 inhibitors in our patients who will probably benefit the most from their use. Low rates of use of renin–angiotensin–aldosterone blockers among patients with CKD, more than 20 years after establishing their benefit in this population, warns against therapeutic inertia that disproportionally affects patients with CKD^34^.

Our study has several limitations. It is treating eGFR using a dichotomous value because we did not have access to patient level data that would allow using individual creatinine or eGFR values. In addition, all outcomes were not reported in all studies by CKD status and definition of cardiovascular and renal outcomes was not uniform across the studies. However, inclusion of 38,949 patients with CKD from 12 trials with different inclusion criteria, representing a diverse population, absence of significant heterogeneity for most outcomes, and similar results in various analyses constitute unique strengths of our meta-analysis. A future meta-analysis using patient-level data will allow for a more granular representation of the effect of renal function on the safety and efficacy of SGLT-2 inhibitors. In such a study, a standardized definition for the renal outcome, such as the composite outcome of 40% GFR decline, ESKD, or renal death as primary renal endpoint, could be used to improve comparability. A patient-level meta-analysis will also allow to report rare side effects, such as euglycemic ketoacidosis, by CKD status at baseline. Another important consideration is inclusion of ethnically diverse populations and people from underserved areas that needs to be addressed in future studies. Finally, although there seems to be a class effect from SGLT-2 inhibitors with respect to their cardiovascular and renal benefits, future studies should proceed with head-to-head comparisons of different SGLT-2 inhibitors.

In conclusion, SGLT-2 inhibitors offer strong protection against cardiovascular and renal outcomes in patients with CKD, with and without diabetes. These results strongly advocate in favor of using these agents in patients with CKD and keeping them until dialysis initiation. The challenge for the nephrology community to widely use this therapeutic option is now ahead.

### Supplementary Information

Supplementary Figures.

## Data Availability

All data generated or analysed during this study are included in this published article and its supplementary information files.
