# Supplementary material for: SGLT-2 inhibitors improve cardiovascular and renal outcomes in patients with CKD: a systematic review and meta-analysis
Source: Sci Rep. 2023 Sep 23;13:15922. doi: 10.1038/s41598-023-42989-z (PMC10517929; doi:10.1038/s41598-023-42989-z)
Supplement: Supplementary file 1 — Supplementary Figures. [file 41598_2023_42989_MOESM1_ESM.pdf]

Supplement

Supplemental Figure 1. Study flow chart

Supplemental Figure 2. Risk-of-bias assessment.

Supplemental Figure 3. Funnel plot of the randomized trials included in this meta-analysis.

Supplemental Figure 4. Forest plot showing the incidence of cardiovascular death or heart failure with SGLT-2 inhibitors compared with placebo in patients with and without chronic kidney disease (CKD).

Supplemental Figure 5. Forest plot showing the incidence of all-cause mortality with SGLT-2 inhibitors compared with placebo in patients with and without chronic kidney disease (CKD).

Supplemental Figure 6. Forest plot showing the incidence of treatment discontinuation with SGLT-2 inhibitors compared with placebo in patients with and without chronic kidney disease (CKD).

Supplemental Figure 1. Study flow chart

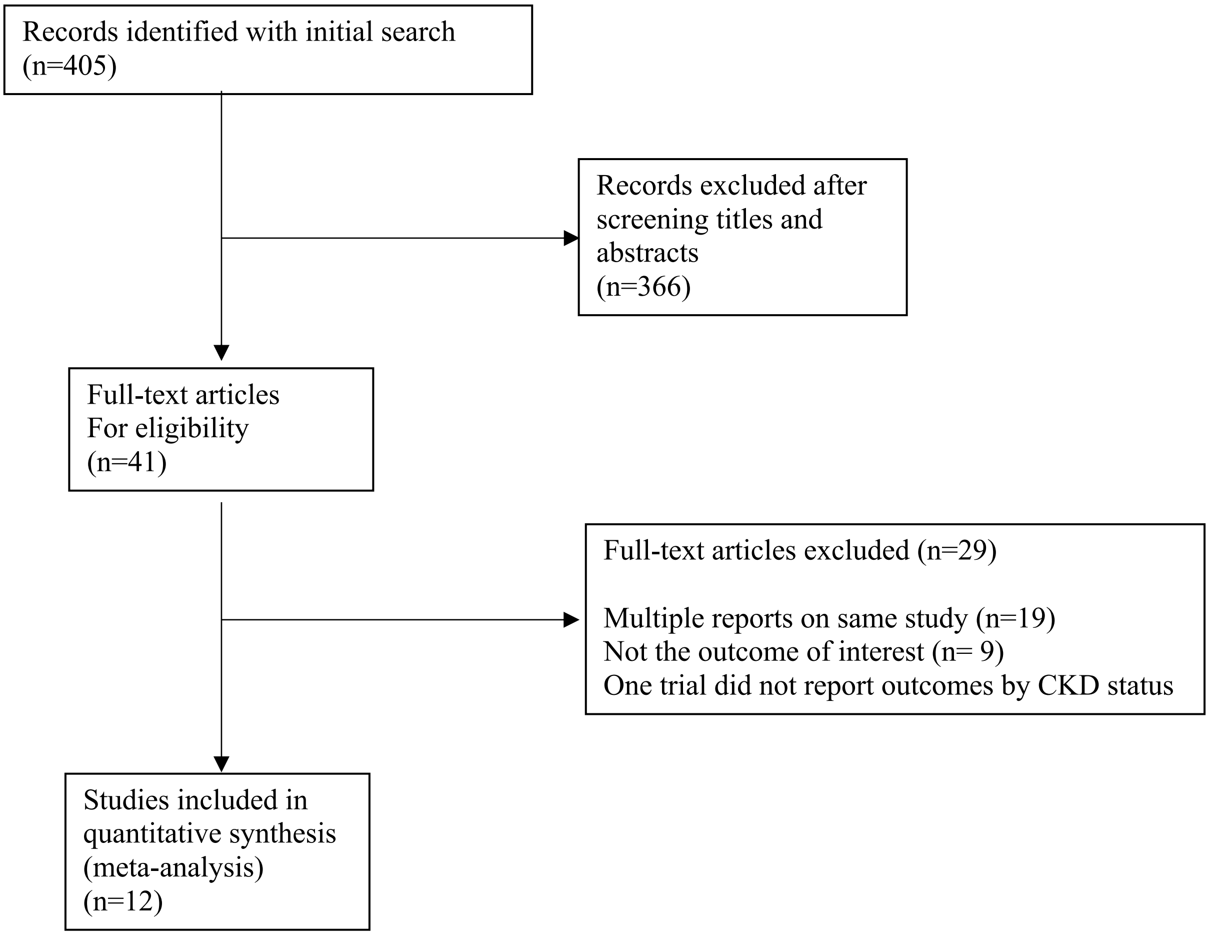

Supplemental Figure 2. Risk-of-bias assessment.

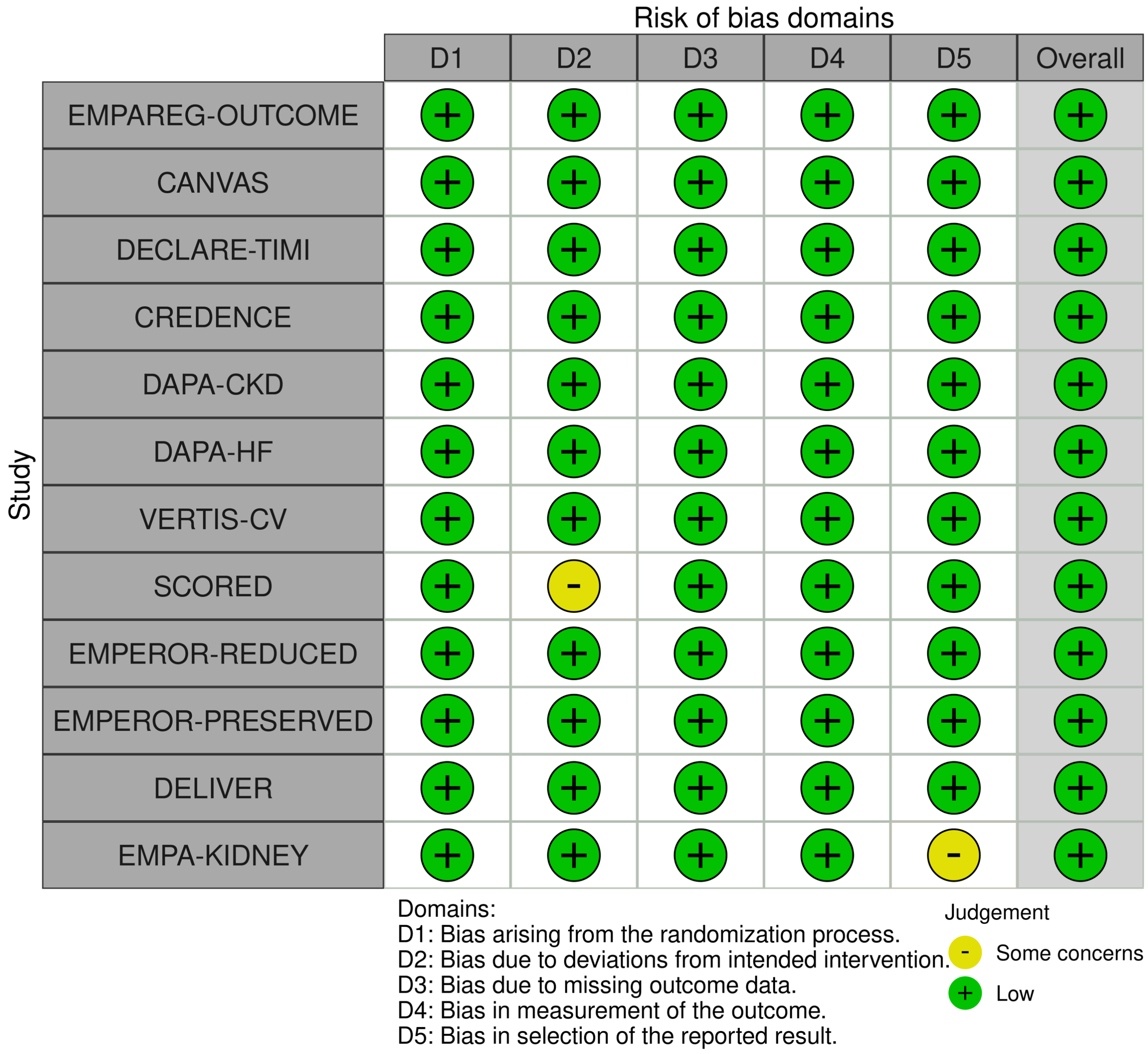

The Cochrane risk-of-bias tool for randomized trials version 2 (RoB2) was used. In this color-coded ranking, green color represents low risk of bias and yellow some concerns.

Supplemental Figure 3. Funnel plot of the randomized trials included in this meta-analysis.

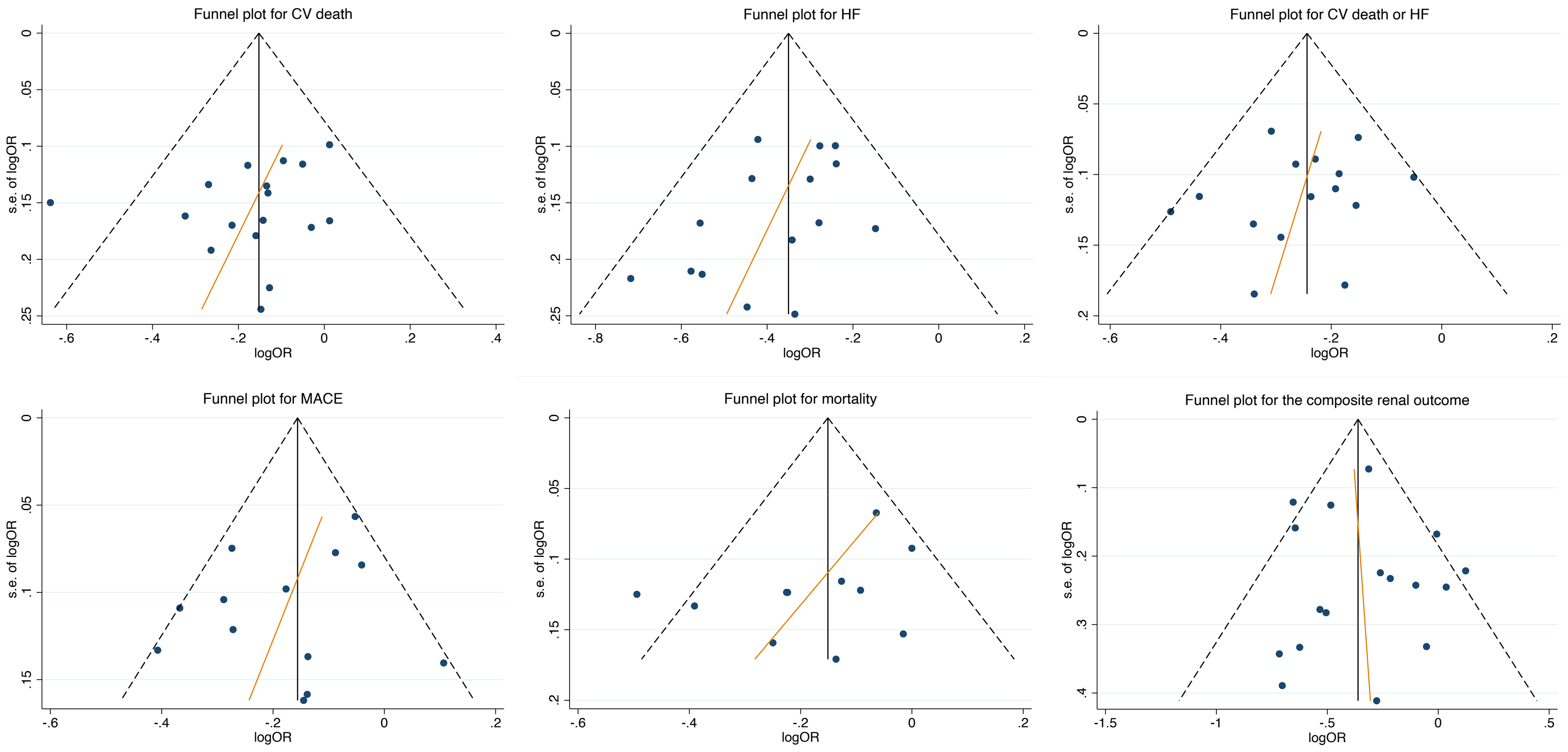

No major publication bias was identified for each of the clinical outcomes.

Supplemental Figure 4. Forest plot showing the incidence of cardiovascular death or heart failure with SGLT-2 inhibitors compared with placebo in patients with and without chronic kidney disease (CKD).

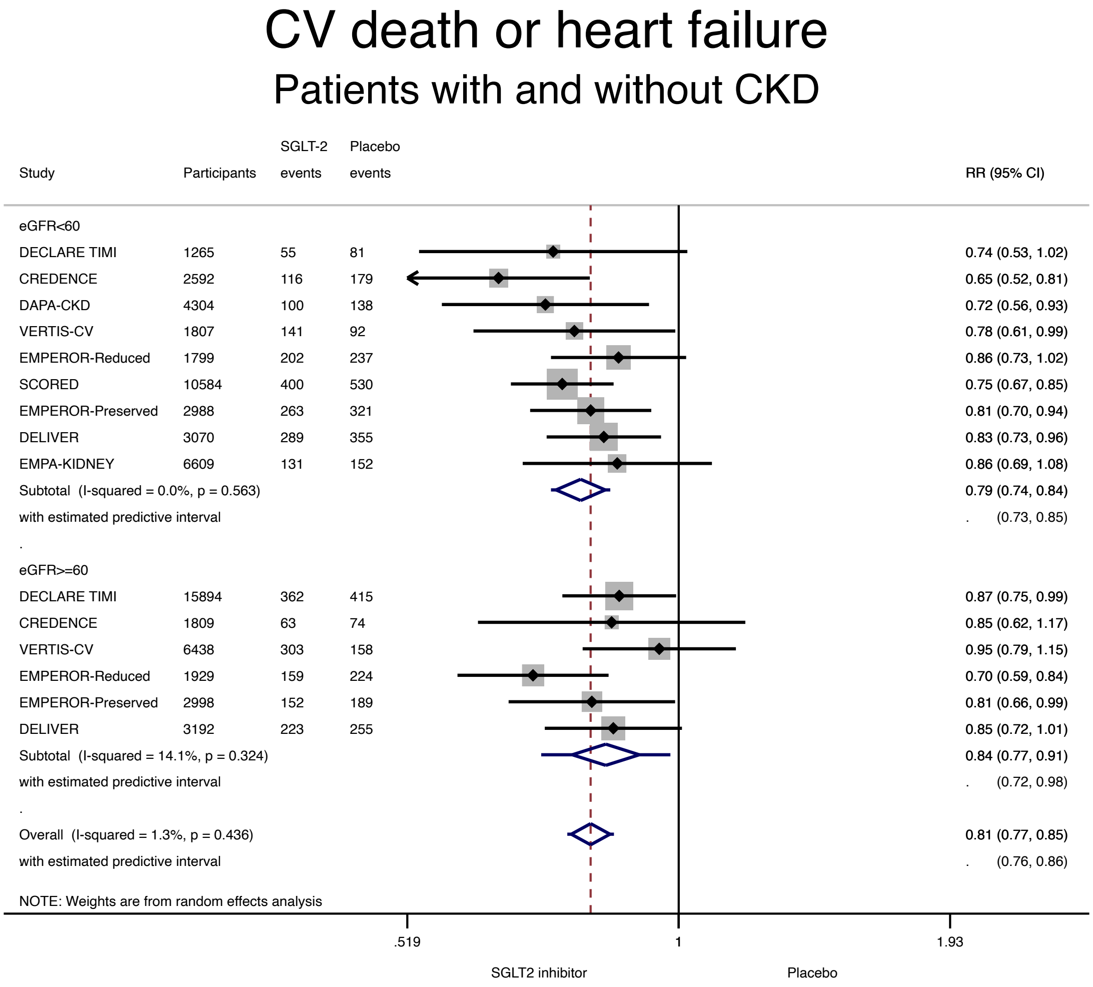

Results are stratified by CKD status. Only results from randomized controlled trials are included. Data are presented as risk ratios (RR) with 95% confidence intervals (95% CI). A lower incidence of cardiovascular death or heart failure is identified with SGLT-2 inhibitors compared with placebo in patients with and without CKD (p for interaction 0.54). A random effects model is used.

Supplemental Figure 5. Forest plot showing the incidence of all-cause mortality with SGLT-2 inhibitors compared with placebo in patients with and without chronic kidney disease (CKD).

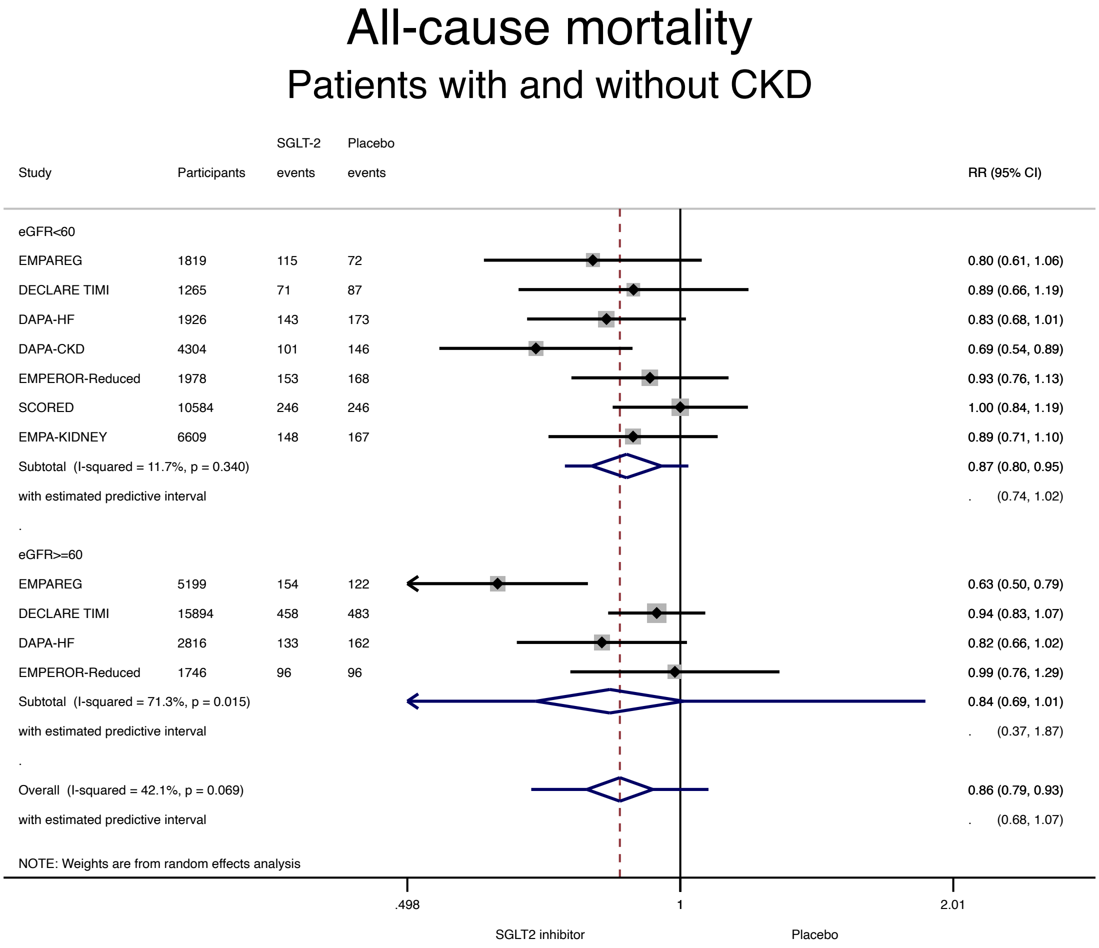

Results are stratified by CKD status. Only results from randomized controlled trials are included. Data are presented as risk ratios (RR) with 95% confidence intervals (95% CI). A lower incidence of all-cause mortality is identified with SGLT-2 inhibitors compared with placebo in patients with CKD but not among patients without CKD (p for interaction 0.79). A random effects model is used.

Supplemental Figure 6. Forest plot showing the incidence of treatment discontinuation with SGLT-2 inhibitors compared with placebo in patients with and without chronic kidney disease (CKD).

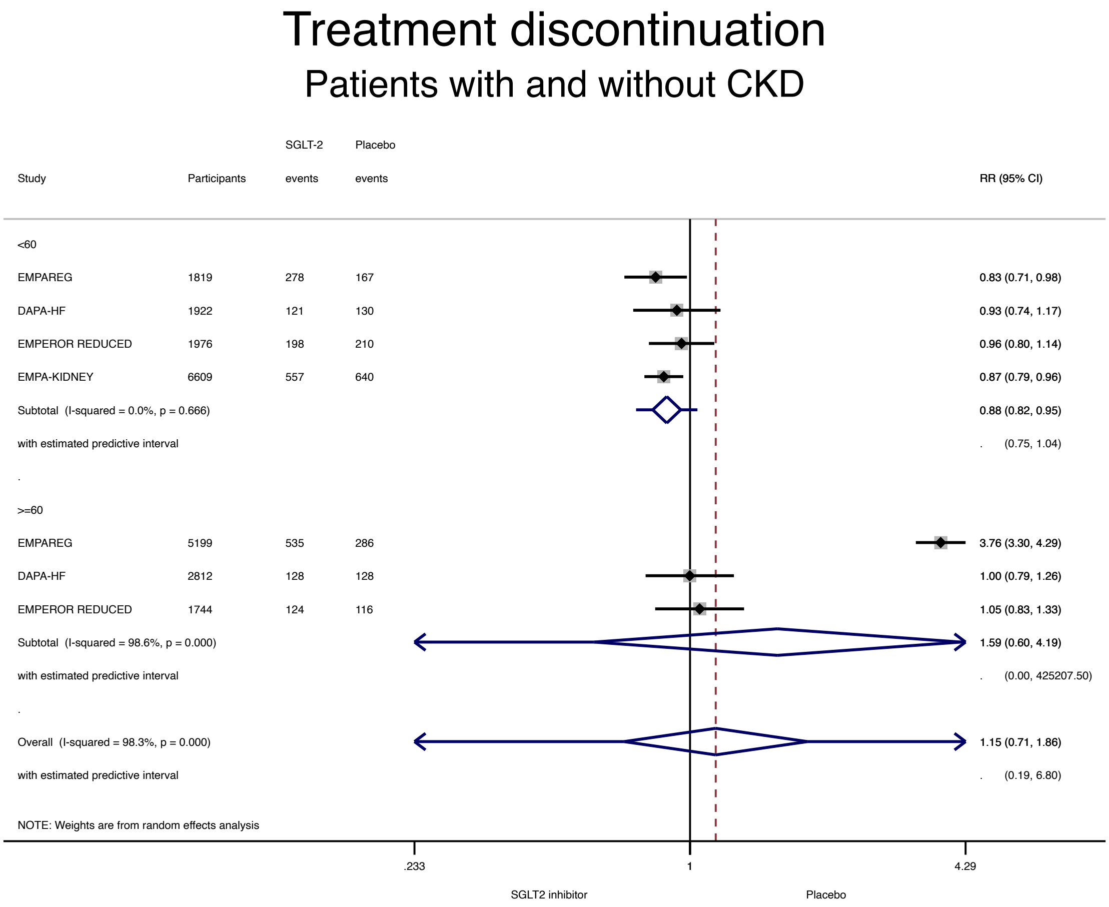

Results are stratified by CKD status. Only results from randomized controlled trials are included. Data are presented as risk ratios (RR) with 95% confidence intervals (95% CI). A lower incidence of treatment discontinuation is identified with SGLT-2 inhibitors compared with placebo in patients with CKD but not among patients without CKD. A random effects model is used.
